# The effects of revised peer-counselor support on the PMTCT cascade of care: results from a cluster-randomized trial in Kenya (the EMMA study)

**DOI:** 10.1186/s12879-023-08246-4

**Published:** 2023-04-25

**Authors:** Bruce A. Larson, Isaac Tsikhutsu, Margaret Bii, Nafisa Halim, Patricia Agaba, William Sugut, Jane Muli, Fredrick Sawe

**Affiliations:** 1 Department of Global Health, Boston University School of Public Health, 801 Massachusetts Avenue, Boston, MA 02118 USA; 2 Kenya Medical Research Institute, Kericho, Kenya/U.S. Army Medical Research Directorate-Africa, Nairobi, Kenya; 3 U.S. Military HIV Research Program, Walter Reed Army Institute of Research, Silver Spring, Maryland, USA; 4 HJF Medical Research International, Kericho, Kenya; 5 Henry M. Jackson Foundation for the Advancement of Military Medicine, Bethesda, MD USA

**Keywords:** HIV/AIDS, Antiretroviral therapy (ART), Prevention of mother-to-child transmission (PMTCT), Mentor mothers, Proportion of days covered (PDC) with medications, Infant HIV testing

## Abstract

**Background:**

This study evaluated the effect of revisions to existing peer-counselor services, called Mentor Mothers (MM), at maternal and child health clinics on medication adherence for women living with HIV (WLWH) in Kenya and on early infant HIV testing.

**Methods:**

The Enhanced Mentor Mother Program study was a 12-site, two-arm cluster-randomized trial enrolling pregnant WLWH from March 2017 to June 2018 (with data collection through September 2020). Six clinics were randomized to continued MM-supported standard care (SC). Six clinics were randomized to the intervention arm (INT = SC plus revised MM services to include more one-on-one interactions). Primary outcomes for mothers were defined as: (PO1) the proportion of days covered (PDC) with antiretroviral therapy (ART) ≥ 0.90 during the last 24-weeks of pregnancy; and (PO2) ≥ 0.90 PDC during the first 24-weeks postpartum. Secondary outcomes were infant HIV testing according to national guidelines (at 6, 24, and 48 weeks). Crude and adjusted risk differences between study arms are reported.

**Results:**

We enrolled 363 pregnant WLHV. After excluding known transfers and subjects with incomplete data extraction, data were analyzed for 309 WLWH (151 SC, 158 INT). A small share achieved high PDC during the prenatal and postnatal periods (0.33 SC/0.24 INT achieved PO1; 0.30 SC/0.31 INT achieved PO2; crude or adjusted risk differences were not statistically significant). In addition, ~ 75% in both study arms completed viral load testing during year two after enrollment, with > 90% suppressed in both arms. For infants, ≥ 90% in both arms had at least one HIV test through study follow up (76 weeks) but testing on schedule according to PMTCT guidelines was uncommon.

**Conclusions:**

While national guidelines in Kenya recommended that all HIV-infected pregnant women take a daily antiretroviral regimen for life following a HIV diagnosis, results presented here indicate that a minor share achieved high medication coverage during the prenatal and postnatal periods analyzed. In addition, adjustments to Mentor-Mother services showed no improvement in study outcomes. The lack of effect for this behavioral intervention is relatively consistent with the existing literature to improve mother-infant outcomes along the PMTCT care cascade.

**Clinical Trial Number:**

NCT02848235. Date of first trial registration 28/07/2016.

**Supplementary Information:**

The online version contains supplementary material available at 10.1186/s12879-023-08246-4.

## Background

The World Health Organization issued recommendations for prevention of mother-to-child- transmission (PMTCT) of HIV in 2000 [1]. By 2011, a global plan was developed for eliminating HIV infections among children by 2015 [2]. As part of the 2011 global plan, 22 countries representing 90% of the world’s HIV-positive pregnant women at the time were prioritized for elimination of mother-to-child transmission [3]. This elimination goal, when not achieved, was then folded into the UNAIDS 2016–2021 fast-track strategy to end AIDS, which set a target of zero new HIV infections for 2020 [4].

Kenya was one of the 22 global plan priority countries where, despite impressive gains, mother-to-child transmission remained stubbornly high some years after the 2011 global plan and the 2014 policy switch to immediate initiation of life-long antiretroviral therapy (ART) for pregnant and breastfeeding women upon an HIV diagnosis [5]. As of 2017, the Kenyan Ministry of Health estimated that 69,500 pregnant women living with HIV (WLWH) needed PMTCT services, with a mother-to-child transmission rate of 11.5% [6]. Based on this information, an additional 8,000 infants were estimated to be newly infected in 2017, with another 61,500 HIV-exposed but uninfected infants.

While PMTCT involves a package of multiple services, rapid initiation of life-long ART for all WLWH, both pregnant and not yet pregnant, has been a key component of PMTCT services since the 2016 guidelines in Kenya [7]. Early initiation of ART, with good adherence and viral suppression, is proven and highly effective for PMTCT [8]. ART also improves the mother’s health during pregnancy and after delivery, which is associated with better health outcomes for HIV-exposed but uninfected infants [9, 10].

Existing literature has documented a range of typical problems implementing PMTCT services, including late presentation for prenatal care and the lack of retention and/or inconsistent visits before and after delivery [11–22]. These issues then contribute to incomplete viral load testing for mothers and HIV testing for HIV-exposed infant. Despite the importance of ART as a component of PMTCT, assessment of ART coverage (initiation and adherence) over key periods in the PMTCT cascade of care and interventions to assess and improve coverage have also been lacking. For example, systematic reviews based on literature published by 2015 identified a limited number of studies evaluating interventions to improve PMTCT service delivery, but ART coverage was not a primary outcome in any of the studies [23, 24]. For later literature, a 2018 meta-analysis of interventions addressing adherence in pregnant women showed fewer than “60% of women were adherent to ART”, although it is difficult to assess this number given that the same review concluded that most of the reviewed studies used self-reporting measures to assess medication adherence without identifying the tools used or if they were validated [25]. Another 2019 systematic review and meta-analysis summed up the literature as: “evidence on the effectiveness of interventions to improve uptake and retention of mothers and infants in PMTCT care is lacking” (p.1 in [26]).

Against this backdrop, the Enhanced Mentor Mother progrAm (EMMA) study was designed to evaluate whether modest revisions to standard services provided by peer counselors (called Mentor Mothers) at maternal and child health (MCH) clinics could improve retention along the PMTCT cascade of care. We report here results for primary and secondary outcomes.

## Methods

### Study design and setting

The EMMA study was a site-randomized, pragmatic clinical trial. A clustered design (at the clinic level) was used because the intervention in the study was a quality-improvement intervention implemented at MCH clinics, rather than at an individual patient level [27]. EMMA was a pragmatic trial because the all PMTCT and Mentor Mother services provided in either study arm were implemented as routine practice by clinic staff [28].

The study was implemented in 12 public MCH clinics within the Kenya Medical Research Institute (KEMRI)/Walter Reed Project (WRP) PEPFAR program (South Rift Valley and Kisumu West). These facilities provided the majority of PMTCT services for HIV-infected pregnant and post-partum women in the region. Sites were stratified into six pairs based on size and location. A simple randomization to standard care or the intervention arm for each pair of clinics was completed by the study Principal Investigator. At each clinic, mothers meeting eligibility criteria and providing written informed consent where then enrolled sequentially overtime at each clinic in the study. After enrollment, other than interactions with the Mentor Mother as part of EMMA activities in the intervention arm, study staff had no contact with study participants.

### Standard care (SC) and intervention (INT) arm procedures

﻿Study enrollment began in 2017, and all study sites provided PMTCT services based on Kenyan guidelines, which included continuation of ART for those already on treatment and immediate eligibility for ART (once-a-day, fixed-dose combination of tenofovir, lamivudine, and efavarivnz) for those not yet on ART, along with additional services outlined in national treatment guidelines [7, 29].

As additional support, beginning in 2012, peer-counselors called Mentor Mothers were integrated into MCH clinics providing PMTCT [30–33]. In general, MCH clinics might have one or two Mentor Mothers depending on clinic size and funding. The national guidelines for Mentor Mothers summarizes the general categories of activities to be completed by Mentor Mothers, which are all facility based [31]. In particular, Mentor Mothers guidelines recommend that at least one, one-on-one, counseling session should occur between a Mentor Mother and her client (HIV-infected pregnant women) over the course of a pregnancy [31]. The six clinics randomized to the standard care arm continued to provide PMTCT services as usual, which included Mentor Mother who were existing clinic staff (not study staff) to all patients at these sites.

The six clinics randomized to the intervention arm continued to provide PMTCT services based on Kenya guidelines, but with two main revisions to the services provided by Mentor Mothers (and see [34] for additional detail). In the intervention arm, rather than one, one-on-one counseling session during pregnancy, the goal was for a Mentor Mother to complete an exit discussion/counseling session with each of her clients at the end of each clinic visit. The purpose of this exit discussion was to review clinical care received at the visit, answer questions, discuss any concerns of the mother, review the schedule for her next visit, and discuss the importance of attending the next visit. The small guide developed for Mentor Mothers in the intervention arm has been published [34]. And second, as part of each discussion, Mentor Mothers were also advised to give each mother the option to receive an automatic text message from Mentor Mother before her next visit to assist with planning for the visit (and to follow up by text, phone, or home visit if late for a clinic visit). Note that Mentor Mothers at the intervention clinics were clinic staff, not study staff, and provided EMMA services to all clients during the study period, not just those enrolled in the study for data access (as discussed below).

### Study population

﻿﻿The study population was all pregnant women with HIV presenting at a study site (MCH clinics) to begin prenatal care. This included women who were newly diagnosed with HIV when presenting for prenatal care (treatment naïve) as well as pregnant women with HIV already on ART (treatment-experienced) when presenting for prenatal care. HIV testing for pregnant women presenting for prenatal care is high in Kenya (> 90% national from 2013; > 95% in 2018) [35, 36]. Inclusion criteria were: aged 18 years or older; pregnant with HIV presenting for prenatal care at a study clinic; and the ability to understand and the willingness to sign/mark a written informed consent document in English, Kiswahili, or Luo during first or second visit for prenatal care at a study site. Exclusion criteria were: they did not intend to receive further prenatal, postnatal, or PMTCT care at the site; and/or were not physically and/or emotionally able to complete the informed consent process.

### Data collection and outcome measures

﻿Follow-up for data extraction was passive, by medical record review only, from the date of an enrolled mother’s first visit for prenatal care up to 72-weeks postpartum (and 76 weeks of age for her infant). Viral load test dates and results for mothers and infant early-infant diagnosis (EID) HIV DNA PCR test dates and results were extracted from the national database maintained by Kenya’s National AIDS Control Program (NASCOP).

The primary outcomes (PO) for mothers as defined in the original study protocol were: the proportion of mothers who received an uninterrupted supply of ART from treatment initiation to delivery (PO1) and from delivery to 24 weeks post-partum (PO2). ﻿To operationalize an “uninterrupted supply”, the proportion of days covered (PDC) with ART was used, with coverage at least 90% considered an uninterrupted supply. PDC is a standard indirect measure of adherence to chronic medications [37–40]. PO1 was further restricted to the last 24 weeks of pregnancy. With both treatment naïve and experienced mothers eligible for study participation, focusing on the date of treatment initiation would have created widely disparate time periods for PO1 [41]. See the Supplemental file for additional detail on measuring these primary outcomes.

The 24-week prenatal and postnatal periods for PO1 and PO2 were informed by Kenyan guidelines and information on time-to-viral-suppression after ART initiation. For PO1, viral suppression within 24 weeks of initiation is highly likely with good adherence [42]. For PO2, the 24-week postnatal window matches the time period for a second infant HIV test in Kenyan guidelines.

The 90% threshold for PO1 and PO2 was chosen for two reasons. First, prior literature suggests that high coverage (e.g., ≥ 95%) is required for long-term viral suppression [43, 44]. And second, other studies have used a similar threshold for evaluating ART coverage [38, 45]. Given that more recent literature suggests lower levels of adherence can achieve viral suppression [43], results of a sensitivity analysis using 80% coverage are also reported.

Study specified secondary outcomes for infant HIV testing were the proportion of infants completing HIV testing at 6, 24, and 48 weeks (+ -4 weeks for each). In addition, the number testing positive are also reported.

Viral load test results over specific time periods were not included as original study outcomes because routine viral load monitoring was introduced in Kenya after the original study protocol was developed but before enrollment began. In short, viral load testing should occur approximately every six months after presenting for prenatal care (given rapid ART initiation for WLWH not on ART when presenting) through cessation of breast feeding [7]. Based on these guidelines, all mothers (those on ART or not yet on ART when presenting for prenatal care) should have at least one viral load test during the first and second year after presenting for prenatal care. Proportions tested within these follow up periods along with viral suppression are reported (< 1000 copies/ml considered virally suppressed).

### Sample size

﻿Each primary outcome is a proportion, and the original sample size (30 subjects per clinic, 360 total, 180 per arm) was developed (using *sampsi* and *sampclus* in STATA) to be adequate to detect at least a 25 percentage-point improvement in each primary outcome between the two study groups (5% significance, 80% power, SC proportion 0.40, intracluster correlation ≤ 0.05; see Table 1 in [34] for full details).

**Table 1:** Baseline characteristics for mothers enrolled in the study

	**Standard Care Arm**		**Intervention Arm**	
Analyzed (number)	**151**		**158**	
*Characteristics at baseline*	**Number**	**Percent**	**Number**	**Percent**
**Age**				
18 to 29	90	60%	74	47%
30 to 39	51	34%	75	47%
40 +	8	5%	5	3%
missing	2	1%	4	3%
**Delivered in a health facility**				
Yes	122	81%	124	78%
No	11	7%	11	7%
missing	18	12%	23	15%
**Weeks gestation**^**a**^				
Less than 20 weeks	32	21%	29	18%
20 to < 30 weeks	63	42%	57	36%
30 + weeks	50	33%	62	39%
missing	6	4%	10	6%
**Treatment status when presenting for prenatal care**				
Naïve (not yet on ART)	45	30%	27	17%
Experienced (on ART)	106	70%	131	83%
**Baseline viral load if treatment experienced at enrollment**^**b**^	106		131	
No test in window	36	34%	71	54%
VL > 1000 copies/ml	2	2%	3	2%
VL ≤ 1000 copies/ml	68	64%	57	44%
**Mentor Mothers new at site**				
Yes	39	26%	56	35%
No	112	74%	102	65%
**Study enrollment during nurses’ strike**				
Yes	112	74%	130	82%
No	39	26%	28	18%
**Study follow up period during nurse strike**				
Yes	112	74%	158	100%
No	39	26%	0	0%

^a^ Weeks gestation: Based on 40-week gestation period and days between day presented for prenatal care and delivery date. If no delivery date, then uses expected delivery date from mother's medical file if it exists

^b^ Viral load testing status if treatment- experienced (+—90 days from first visit for prenatal care)

### Data analysis

At the individual level, primary outcomes are dichotomous variables. For each study arm, these dichotomous outcomes are summarized as proportions. All analyses are by modified intention to treat; subjects were excluded who were known by the clinic to have transferred to other clinics, and subjects were excluded with known incomplete data collection (see the Supplemental file for additional information). For the infant HIV-testing secondary analyses, mother-infant pairs were then excluded with adverse birth outcomes (e.g., miscarriage, still birth, neonatal death, mother death).

A linear probability model was used to estimate risk differences between study arms [46]. We report crude risk differences and adjusted risk differences, with the adjusted model including a limited set of baseline characteristics of the mother and study clinic [47]. STATA *boottest* was used to adjust for clustering and for the small number of clusters in the study [48, 49].

## Results

### Enrollment

Enrollment was planned to be staggered across the 12 sites beginning in mid-March 2017, with enrollment at all sites to begin quickly after March 2017. However, issues with funding paused enrollment in April 2017 and then enrollment and follow up were further affected by the national nurses’ strike during June to November 2017 and presidential elections in August 2017. Due to these issues, enrollment began at sites through December 2017. See the supplemental file Table S1 for a summary of these issues by site.

**Fig. 1:**
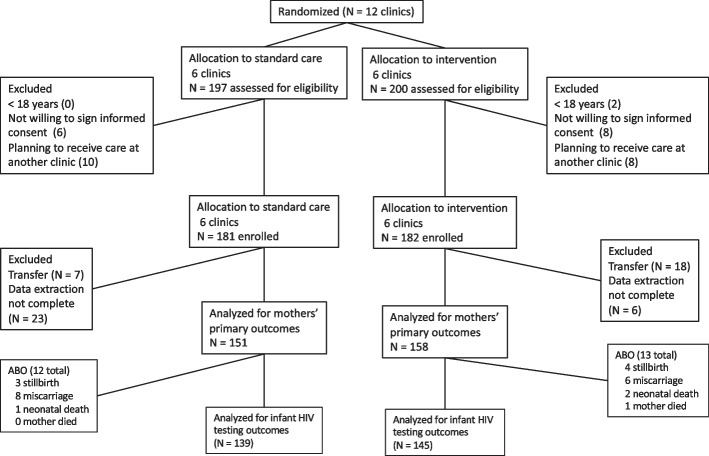
EMMA study CONSORT Diagram

As summarized in Fig. 1, the study assessed 397 women, and enrolled 363 women. Enrolled women who transferred to another clinic were then excluded from analysis (25 total). In addition, for analysis, 29 enrolled women were excluded if final data extraction was not completed due to the various study delays noted in the supplemental file Table S2. Among the 309 mothers included in the primary outcomes analysis (excluding transfers and incomplete data extraction), 25 mothers experienced some form of adverse birth outcome (ABO). For infant HIV-testing outcomes, the final sample analyzed is then 284.

### Baseline characteristics

Data on mothers’ characteristics at enrollment (Table 1) were limited to information consistently recorded in medical records. From Table 1, the intervention arm, compared to the standard care arm, was somewhat older and presented later for prenatal care. The intervention arm included proportionately more treatment-experienced mothers, but with less coverage of viral load testing when presenting for prenatal care (as recommended in guidelines). Proportionately more mothers in the intervention arm received care at clinics where mentor mothers were new staff at the clinics (no experience with mentor mothers before the study). More intervention arm mothers enrolled during the nurses’ strike. Two standard arm clinics were the last clinics to begin the study, so enrollment and all follow up was after the strike ended.

### Primary outcomes for mothers

During the final 24 weeks of pregnancy, the mean PDC was 0.63 in standard care and 0.55 in the intervention arm. During just the final 12 weeks of pregnancy, these proportions improved for the intervention arm (0.64 SC; 0.69 INT). In addition, > 95% of women not already on ART then initiated treatment before delivery.

As shown in Table 2, a third or fewer women achieved high ART coverage (≥ 0.90) during the final 24 weeks of pregnancy (PO1). The crude results for PO1 are essentially the same whether based on the full sample (Crude) or the sample limited to those with data on baseline covariates used in the adjusted model (Crude 2). These crude risk differences are also very similar to the adjusted risk difference, all three models suggesting no statistical difference in this outcome between study arms.

**Table 2:** Proportion of days covered (PDC) with antiretroviral medications^a^

Outcome	SC	INT	RD (Crude)^a^	RD (Crude 2)	RD (Adjusted)
			**(95% CI)**	**(95% CI)**	**(95% CI)**
	***N* = 151**	***N* = 158**	***N* = 309 (151/158)**	***N* = 290 (144/146)**	***N* = 290 (144/146)**
PO1: At least 0.90 PDC during last 24 weeks of pregnancy at least 0.90	0.32	0.25	-0.07	-0.07	-0.05
			(-0.223, 0.063)	(-0.217, 0.049)	(-0.176, 0.095)
PO2: At least 0.90 PDC first 24 weeks postpartum	0.30	0.31	0.01	0.02	0.12
			(-0.145, 0.159]	(-0.154, 0.171)	(-0.046, 0.284)

^a^ RD = Risk difference. Crude uses the full sample (309 for mother outcomes, for infant HIV testing outcomes). Crude 2 excludes those without mother age or weeks gestation at the first ANC visit (used in the adjusted model). Adjusted reports risk differences adjusted for baseline covariates: mother's age, weeks gestation at first ANC visit, Mentor Mothers new at the site, site follow up period overlapped with nurses strike

During the first 24 weeks postpartum, the mean PDC was 0.69 in both study arms (and median approximately 0.80). As with PO1, about a third of women achieved high ART coverage in the first 24 weeks postpartum (PO2). While the crude results (Crude or Crude 2) are similar with no difference between study arms, the adjusted RD is substantially larger (but not statistically different), which suggests possible confounding from imbalance in baseline covariates.

### Secondary outcomes for infants

Almost all infants in the analysis were identified in the NASCOP early infant HIV testing database with at least one test through 76 weeks after birth (SOC = 133/139 = 0.96 INT = 133/145 = 0.92). While the study was not powered to address rates of transmission, of those infants identified with any test, a total of 5 tested positive (2 SC, 3 INT), of whom four had two positive tests and one only had one positive test (and no further tests for confirmation).

From Table 3, the majority of infants received the six-week test on schedule (S4). Somewhat fewer infants received the 24-week HIV test on schedule (S5), and few infants received the 48-week HIV test on schedule (S6). While the crude and adjusted results show that somewhat fewer infants completed HIV testing in the intervention arm compared to standard care (at 6, or 12, or 24 weeks), the risk differences are not statistically different from zero at a 5% level. The adjusted model results, when compared to the crude results, suggest possible confounding from imbalance in baseline covariates.

**Table 3:** Infant HIV testing according to national guidelines^a^

Outcome Description	SC	INT	RD (Crude)	RD (Crude 2)	RD (Adjusted)
			**(95% CI)**	**(95% CI)**	**(95% CI)**
	***N* = 139**	***N* = 145**	***N* = 284 (139/145)**	***N* = 276 (136/140)**	***N* = 276 (136/140)**
S4: Infant HIV test at 6 weeks (+ -4 weeks)	0.77	0.70	-0.07	-0.07	-0.13
			(-0.193, 0.036)	(-0.194, 0.041)	(-0.294, 0.019]
S5: Infant HIV test at 24 weeks (+ -4 weeks)	0.59	0.52	-0.07	-0.07	-0.15
			(-0.272, 0.126)	(-0.273, 0.136)	(-0.428, 0.111)
S5: Infant HIV test at 48 weeks (+ -4 weeks)	0.13	0.10	-0.03	-0.03	-0.06
			(-0.173, 0.010]	(-0.179, 0.105]	(-0.220, 0.113)

^a^ Using the same approach for the Crude 2 and Adjusted models as in Table 2

An original primary study outcome was infant HIV testing at 72 weeks (+—4 weeks). While no infants received the early-infant HIV test during this period (based on the NASCOP database), these older infants would have received a rapid test at their clinic, and these results would not be included in the NASCOP database (and as summarized in the Supplemental file, the study ended before complete data at 72 weeks could be extracted from medical records at clinics and entered into the study database).

### Viral load testing for mothers

From Table 4, about 75% of women in each study arm received at least one follow up viral load test during the first year after presenting for prenatal care (technically day 91–365 after the first prenatal care visit; any test + -90 days from the first visit was considered a baseline visit). Among those tested during year one, 110 of 117 (94%) in the SC arm and 112 of 120 (93%) in the INT were suppressed (based on VL < 1000 copies/ml). Proportions VL tested during year two were similar to rates for year one (0.74 SC, 0.72 INT). Among those tested during year two, 106 of 112 (95%) in the SC arm and 108 of 114 (95%) in the INT arm were suppressed. Crude and adjusted risk differences are not statistically different from zero at a 5% level.

**Table 4:** Viral load testing during Year 1 and Year 2 after presenting for prenatal care

Outcome (exploratory)	SC	INT	RD (Crude)	Crude 2	Adjusted
			**(95% CI)**	**(95% CI)**	**(95% CI)**
	***N* = 151**	***N* = 158**	***N* = 309 (151/158)**	***N* = 290 (144/146)**	***N* = 290 (144/146)**
Viral load testing Year 1 (91–365 days after first prenatal care visit)	0.77^a^	0.76	-0.02	-0.01	-0.10
			(-0.234, 0.196)	(-0.208, 0.178)	(-0.397, 0.158)
Viral load testing Year 2 (366—730 days after first prenatal care visit)	0.74	0.72	-0.02	0.02	-0.06
			(-0.171, 0.138)	(-0.134, 0.163)	(-0.195, 0.060)

^a^ Proportions. If two or more tests completed during each year, viral suppressed was based on results for the last test within each year

### Sensitivity analysis (80% PDC)

Not surprisingly, if the threshold for defining high coverage is reduced, the proportion of women in both study arms achieving the threshold increases during both the 24-week prenatal and postnatal periods. Table 5 summarizes results for an 80% threshold (PDC ≥ 0.8). These numbers continue to gradually increase with lower thresholds down to 50%.

**Table 5:** Sensitivity analysis with 80% PDC threshold

Sensitivity analysis	SC	INT	RD (Crude)
			**(95% CI)**
	***N* = 151**	***N* = 158**	***N* = 309 (151/158)**
At least 0.80 PDC last 24 weeks of pregnancy	0.42	0.40	-0.02
			(-0.188, 0.142)
At least 0.80 PDC first 24 weeks postpartum	0.50	0.47	-0.03
			(-0.210, 0.119)

## Discussion

The EMMA study was a pragmatic, two-arm, cluster-randomized trial in western Kenya examining the effects of two adjustments to Mentor Mother services to support PMTCT services for pregnant women living with HIV and presenting for prenatal care. Potential ART adherence for mothers, based on PDC with antiretroviral medication, and rates of infant HIV testing according to guidelines were not different between study groups.

The lack of impact for this behavioral intervention is relatively consistent with a now existing and large literature evaluating strategies to improve service delivery and mother-infant outcomes along the PMTCT care cascade that did not exist when the study was developed (see, e.g. [23–26, 50–52]. for reviews of such literature; and see [11, 53] for additional examples). In short, PMTCT guidelines during the study period (enrollment beginning in 2017) had already incorporated multiple, previously studied improvements in PMTCT care. These included: (1) treatment-for-all so that delays based on CD4 testing were already eliminated; (2) ART services integrated into maternal and child health clinics as part of routine PMTCT services, so women did not have to attend a different clinic her HIV care; (3) treatment based on a single pill daily; and (4) national guidelines for clinic-based peer counselors (Mentor Mothers) already existed, although implementation was not complete at all study clinics until the study began. In addition, facility-based delivery was the norm in study participants, initiation of additional PMTCT services such as infant prophylaxis was likely though not evaluated as part of the study. Evidence for additional services or interventions to be added to this core package of PMTCT services, based on existing literature remains limited. Nonetheless, going forward substantial room for improvement in medication coverage during and after pregnancy continues to exist.

The EMMA intervention was designed as a low to no incremental cost intervention that was a modest addition to the full package of PMTCT services outlined above and that could be implemented as routine practice outside of a study setting. In comparison, for example, the MOTIVATE trial completed also in western Kenya evaluated a more complex package of services provided by community-based Mentor Mothers (cMMs), including up to 13 home-based visits during prenatal and postnatal care, with about 75% of the intervention arm having 8 or more home visits (hired by the study, along with additional study nurses providing support to cMMs) [11]. Even with this more intensive intervention, retention at 12 months postpartum (based on a clinic visit between month 9 and 15) was not different between study arms. The WiseMama trial is another example of a complex package of services (phone required and provided if needed, daily text message reminders, direct follow up if clinic visits missed (both study arms), cash payments for clinic visits, and technology for monitoring adherence). After excluding about 20% of patients enrolled during the pre-randomization period (poor reception, patient’s plan to discontinue use of the Wisepill device, missed scheduled one-month clinic visit), adherence as defined for the study (opening Wisepill device within 2 h of a daily chosen time) was modest (roughly 50% of days during the final intervention month – month 3 postpartum), with no difference between study arms.

Retention in the MOTIVATE trial and adherence in the WiseMama study were improved in both studies for per-protocol analyses, but a large share of patients were excluded in the per-protocol analyses, in which case self-selection complicates interpretation of the per-protocol results (MOTIVATE: about 50% of intervention groups excluded; WiseMama: about 65% excluded in intervention arm and 54% excluded in standard care arm). Fidelity of implementing the EMMA intervention, unfortunately, cannot be evaluated at this time. The study was designed to be implemented under routine conditions with little to no direct incremental costs. Due to technical failures experienced when using a free text messaging application, data on text messaging between Mentor Mothers and clients was lost. In addition, due to funding issues, the study team did not complete data entry from the study’s Mentor-Mother/patient visit record form (specifically data on the completion of one-on-one sessions at clinic visits along with SMS or phone call reminders based on consent at each visit). Given the study was implemented as part of routine services at the intervention clinics by clinic staff (not study staff), less than perfect implementation is to be expected. The limited data that are available suggest that study participants generally consented to text message or phone call follow ups, but few women consented to follow up visits at home if late for clinic visits.

The study faced important limitations beyond those already discussed (funding, inability to complete data collection for ART coverage after 24 weeks postpartum or for infant HIV testing at 18 months, inability to assess implementation fidelity). Study data for medication coverage (visit dates, quantities and types of medications prescribed), and date of delivery came from the mother’s paper-based medical file at the clinic. Data from paper-based clinical files may contain errors or omissions (including possible missing information for clinic visits). As clinics in Kenya and elsewhere develop and expand electronic pharmacy records, such potentially easier to access and better-quality data will allow for easier and more precise estimates of medication coverage over time. Data for viral load testing and early infant HIV testing (dates and results) were extracted from the national testing database maintained by NASCOP. Matching clinic-based IDs for mothers and infants to those in the NASCOP database was laborious because of the different number formats and non-unique identification numbers in the NASCOP database. In the future, with standardization of identification numbers used at clinics, laboratories, and databases, follow up over time should continue to improve.

Despite the lack of effectiveness of the EMMA intervention observed in this study, the basic results for both study groups provide useful information for future evaluation of PMTCT programs and outcomes. First, as electronic medical and pharmacy records become more common, obtaining data to measure the proportion of days covered with medications will become substantially easier to use for evaluation purposes rather than, or in addition to, simple measures of retention at some point in time. Second, women presented relatively late in their pregnancy for prenatal care (20 + weeks gestation), consistent with prior literature [54, 55]. Third, the majority of women living with HIV and presenting for prenatal care already knew their status and were on ART, which implies prior studies enrolling only treatment naïve women only address a small share of the target population for PMTCT services (e.g. [53]). And fourth, an important share of women on ART when presenting for prenatal care did not receive a viral load test (34% in standard care arm; 54% in intervention arm). These rates were better, however, than the 73% reported in another study from Kenya [56].

Also going forward, the result presented here highlight that a significant share of “potential PMTCT” time during a pregnancy occurs before the first prenatal visit. Given that an important share of women present late for prenatal care (mean/median presentation at 24 + weeks gestation remains common with a large share presenting after week 30 weeks [11, 56]), strategies to support presenting earlier for prenatal care would perhaps make it easier for women to receive/accept PMTCT services. Given that a large share of women presenting for prenatal care know their HIV status and are already on ART, documenting viral suppression or not when presenting for care remains crucial for identifying the subset with either existing adherence issues or possible drug resistance.

## Conclusions

This evaluation of an adjustment to clinic-based services provided by Mentor Mothers did not improve the main and secondary study outcomes based on proportion of days covered with medications for mothers and coverage of infant HIV testing. With better coverage of viral load status for women on ART presenting for prenatal care, PMTCT services can be targeted specifically to three primary and fundamentally different subsets of women presenting for prenatal care: (1) on ART and virally suppressed, which is likely to be a relatively large share of the total; (2) on ART but not suppressed (likely to be a small share); and (3) not on ART when presenting, which in effect includes those never on treatment and those previously on treatment (which may or may not be ascertained). These three subsets, and documentation of services received, will also support investigations into the health and development outcomes of HIV-exposed but uninfected infants [9, 10, 57–62]. The approach applied in this study can allow nuanced measures of infants’ exposures based on mother’s medications and coverage and HIV infection (based on viral load testing and results) during multiple periods along the PMTCT cascade of care.

## ﻿Availability of data and materials

Data extracted from routine medical records are owned by the study sites cannot be made publicly available by the authors. The analytic dataset is available from the KEMRI Scientific and Ethics Review Unit (SERU) for researchers who meet the criteria for access to confidential data and have approval from the KEMRI SERU (seru@kemri.org). The corresponding author can assist with such requests.

## Supplementary Information

**Additional file 1: Table S1.** Enrollment period by site with key external events. **Table S2.** Enrollment by site.
