# Supplementary material for: The effects of revised peer-counselor support on the PMTCT cascade of care: results from a cluster-randomized trial in Kenya (the EMMA study)
Source: BMC Infect Dis. 2023 Apr 25;23:257. doi: 10.1186/s12879-023-08246-4 (PMC10127503; doi:10.1186/s12879-023-08246-4)
Supplement: Supplementary file 1 — Additional file 1: Table S1. Enrollment period by site with key external events. Table S2. Enrollment by site. [file 12879_2023_8246_MOESM1_ESM.docx]

**Supplementary file**

This supplemental file contains three sections: (1) additional information on measuring the proportion of days covered with ART; (2) key external events that occurred during study enrollment and follow up, with final enrollment numbers by site; and (3) summary of Mentor Mother responsibilities, training, and additional training for the MMs at the intervention sites.

**1. Estimating the proportion of days covered with ART**

To measure the proportion of days covered (PDC) for each primary outcome, we used the PMTCT-ACT tool developed previously [[1](#_ENREF_1)]. For treatment-naïve study subjects, her MCH clinic records covered her complete history of ART services, permitting direct estimation of PDC.

For study subjects already on ART when presenting for prenatal care (treatment-experienced; n = 237), however, her medical records were located at another clinic before she presented for prenatal care (typically either an HIV-specific or more general outpatient clinic). The study did not have the ability to access the complete history of ART services (potentially multiple years) for these treatment-experienced subjects. To address this issue, we proceeded as follows. Beginning in 2016, Kenya’s guidelines recommended that pregnant women presenting for prenatal care and already on ART for at least six months should receive a viral load test to assess viral suppression ﻿as soon as pregnancy is confirmed [[2](#_ENREF_2)]. This recommendation was then revised to a test for those on ART for at least three months [[3](#_ENREF_3)].

For treatment-experienced study subjects (on ART at their first visit), we identified a viral load test for that patient in the national viral load testing database managed by the National AIDS and STI Control Program (NASCOP) that was nearest to the first visit date for prenatal care. If she had no viral load test during the 90-day window, she was assumed to have 0 days of ART until her first supply at the MCH clinic. If she had a viral load test within 90 days of her first visit (+ 90 days), and she was virally suppressed (defined as < 1000 copies/ml), we assumed she had 100% ART coverage until the date of her first supply of ART at her MCH clinic (n = 125).

If she had a test in the 90-day window but was not suppressed (> 1000 copies/ml), we assumed she had 0% coverage (n = 5).

For the remaining study subjects on ART when presenting for prenatal care who did not receive a baseline viral load test (n = 107), two were lost to study follow immediately after enrolling (so 0% ART coverage during both the prenatal and postnatal periods). Another subset (n = 24) presented for ANC at least 24 weeks before delivery, so complete information was available on ART medications for this group. In the end, about one third of study subjects who were on ART when presenting for prenatal care and did not have a baseline viral load test (81/237), presented for prenatal care within 24 weeks of delivery. For this group, we assumed 0 days covered with ART from week 24 before delivery (168 days) until the date of her first ART supply at the MCH clinic.

This approach is very conservative for treatment-experienced mothers without a guideline-recommended viral load test, given that they likely had some level of ART coverage during this pregnancy period. This approach is also conservative for the potentially few mothers (< 5) who had a viral load result and had good adherence, but were not suppressed because of, for example, unidentified drug resistance. As a sensitivity analysis for measuring PDC for PO1, we considered 50% coverage for treatment-experienced pregnant women without a viral-load test within the + 90 window specified above or tested but not suppressed (n = 86 total). Results for PO1 were unchanged for this sensitivity analysis (PDC improved somewhat for these women but did not push them over the 90% threshold, with no difference between study groups).

Because a delivery date does not exist for women with an adverse birth outcome (SC = 12, INT = 13), they are assumed to have 0% ART coverage for both primary outcomes for the results presented in Table 2 and Table 5. These results are not sensitive to this assumption (i.e., the proportions reported in Table 2 increase slightly if these 25 were excluded from the analysis).

Two secondary outcomes originally included in the study protocol (PDC between weeks 24 – 48 and 48 – 72) could not be evaluated because study follow up and completion was impacted by unplanned events (discussed briefly in the results section).

**2. Summary of key external events that occurred during study enrollment and follow up**

The 12 study clinics provided the majority of PMTCT services for HIV-infected pregnant and post-partum women in the region when the study was developed. The clinics are geographically located in Kisumu, Siaya, Kericho, Nandi, Bomet, and Narok counties. Kisumu and Siaya are counties with relatively high numbers of new HIV infections estimated among children (600+ in each county in 2017), while numbers in the remaining four counties were around 100 each in 2017.[[4](#_ENREF_4)]

Table S1 summarizes the enrollment period for each study site and includes three types of external events that delayed enrollment and/or likely affected services provided at the clinics and patient attending clinic visits. First, study enrollment and follow up overlapped with a national nurses’ strike during June 2 to November 5, 2017. These strikes disrupted delivery of medical care in Kenya during these time periods, as evidenced by substantially fewer admissions during the strike period, substantially lower rates of immunized infants during the strike periods, and higher deaths [[5-8](#_ENREF_5)]. This nurses’ strike overlapped with the enrollment and follow period at nine sites, only the follow up period at one site, and did not overlap with either period at two sites. Given the widespread reporting on the strikes, these strikes could have had two effects on patients at the clinics: (1) pregnant women may have delayed presenting for prenatal care during the strike period to avoid, or at least put off, problems with receiving care during the strike; and (2) those enrolled may have missed clinic visits because either they did not want to return to an understaffed clinic or they returned but did not receive care that day because of understaffed clinics. Enrollment was delayed by 4 months at 9 sites due to the funder and then an additional month at 6 sites due to concerns about violence around the 2017 presidential elections.

In April 2018, the study team then learned that the study was approved for a final year of funding, but funding would likely end as of September 2019. At this point, the decision was made to allow sites to enroll beyond 30 to achieve the target sample size of 180/180 by study arm as quickly as possible. The goal here was to: (1) maximize the number of mothers who could complete their prenatal period and at least 28 weeks of follow postpartum; and (2) to have adequate time for study staff to complete data extraction from clinical records, enter data to the electronic database, and complete basic data quality control prior to data analysis. As summarized in Table S2, the study enrolled 363 women (181 SC, 182 INT). Enrolled women were then excluded from analysis because they transferred during the study follow up period (7 SC, 18 INT) or because funding for the study ended before final data extraction could be completed for these subjects (23 SC, 6 INT).

**Table S1. Enrollment period by site with key external events**

| **Site Number** | **Study Arm** | **Enrollment begins^/1^** | **Enrollment ends** | **Days for enrollment** | **Nurses strike during study^/2,3^** | **Mentor Mothers in place prior to the study** |
| --- | --- | --- | --- | --- | --- | --- |
| **1** | EMMA | 17-Mar-17 | 31-May-17 | 75 | FU | Yes |
| **2** | SC | 21-Mar-17 | 27-Apr-18 | 402 | E, FU | Yes |
| **3** | EMMA | 27-Mar-17 | 29-Nov-17 | 247 | E, FU | Yes |
|  | **PEPFAR Study Pause (no new sites, new enrollment, other study activities)** | | | | | |
| **4** | EMMA | 2-Aug-17 | 30-Apr-18 | 271 | E, FU | Yes |
| **6** | SC | 3-Aug-17 | 2-May-18 | 272 | E, FU | Yes |
| **5** | EMMA | 3-Aug-17 | 1-Mar-18 | 210 | E, FU | Yes |
|  | **No new sties open in preparation for national elections** | | | | | |
| **7** | SC | 10-Sep-17 | 30-May-18 | 262 | E, FU | Yes |
| **10** | EMMA | 10-Oct-17 | 27-Feb-18 | 140 | E, FU | No |
| **8** | SC | 13-Oct-17 | 30-Apr-18 | 199 | E, FU | Yes |
| **9** | EMMA | 18-Oct-17 | 12-Jan-18 | 86 | E, FU | No |
| **11** | SC | 8-Nov-17 | 29-May-18 | 202 |  | No |
| **12** | SC | 4-Dec-17 | 12-Jun-18 | 190 |  | No |

1. Doctor strike between December 5, 2016 and March 14, 2017.

2. Nurses strike during June 5 - November 2, 2017.

3. FU = subject follow up affected by nurses’ strike at the site; E = enrollment period also affected by nurses’ strike.

**Table S2. Enrollment by site**

| Site Number | Study arm^/1^ | Number enrolled in the study | Excluded (early transfer) | Excluded (data extraction not complete) | **Number analyzed for mothers' primary outcomes** | Included but ABO (no date of birth)^/2^ | Number analyzed for infant HIV testing | Included but no data (no date of birth)^/3^ | Analyzed with DOB |
| --- | --- | --- | --- | --- | --- | --- | --- | --- | --- |
| 11 | SC | 28 | 1 | 0 | 27 | 4 | 23 | 0 | 23 |
| 6 | SC | 40 | 3 | 1 | 36 | 1 | 35 | 0 | 35 |
| 8 | SC | 40 | 1 | 1 | 38 | 2 | 36 | 1 | 35 |
| 2 | SC | 34 | 2 | 6 | 26 | 3 | 23 | 2 | 21 |
| 7 | SC | 18 | 0 | 6 | 12 | 1 | 11 | 0 | 11 |
| 12 | SC | 21 | 0 | 9 | 12 | 1 | 11 | 0 | 11 |
|  | **Total SOC** | **181** | **7** | **23** | **151** | **12** | **139** | **3** | **136** |
| 10 | INT | 30 | 2 | 0 | 28 | 1 | 27 | 0 | 27 |
| 3 | INT | 32 | 4 | 4 | 24 | 1 | 23 | 2 | 21 |
| 1 | INT | 30 | 2 | 0 | 28 | 5 | 23 | 1 | 22 |
| 9 | INT | 30 | 2 | 0 | 28 | 2 | 26 | 0 | 26 |
| 5 | INT | 30 | 7 | 0 | 23 | 3 | 20 | 0 | 20 |
| 4 | INT | 30 | 1 | 2 | 27 | 1 | 26 | 0 | 26 |
|  | **Total INT** | **182** | **18** | **6** | **158** | **13** | **145** | **3** | **142** |

^/1^ SC = standard care arm; INT = intervention arm.

^/2^ ABO = adverse birth outcome (see Figure 1 for specific types).

^/3^ For these study subjects, no data were found to document either an adverse birth outcome or the date of birth for the infant.

**3. Mentor Mother Training**

The Kenya Mentor Mother Program guidelines outlines the main requirements for becoming a Mentor Mother (MM), main responsibilities, and pre-service training.[[9](#_ENREF_9)] To qualify to become a MM, a candidate must be an HIV-positive mother with recent PMTCT experience, at least Standard 8 level of education, had disclosed HIV status to at least one person within her household, and lives in the local community. MMs complete an initial two-week pre-service training program and then additional follow up/review training at least annually. MMs have several key responsibility areas, outlined in the KMMP guidelines, such as group education and support activities (see p.40 in [[9](#_ENREF_9)]).

In addition, the KMMP guidelines recommend that MMs have at least one “one-on-one” counseling session with *HIV-negative women* during pregnancy. With the EMMA intervention sites, a main difference from standard care was the incorporation of brief one-on-one sessions (intended as an exist interview/discussion) with *HIV-positive women during and after pregnancy*.[[10](#_ENREF_10)] And [[10](#_ENREF_10)] for additional details of the EMMA intervention along with the brief Mentor Mother Guide used by the MMs at the intervention sites. As pre-service training for the intervention sites, MMs participated in an additional two-day training session for implementing this revised package of services.

**References**

1. Larson BA, Halim N, Tsikhutsu I, Bii M, Coakley P, Rockers PC: **A tool for estimating antiretroviral medication coverage for HIV-infected women during pregnancy (PMTCT-ACT)**. *Global Health Research and Policy* 2019, **4**(1):29.

2. National AIDS and STI Control Programme: **Guidelines on Use of Antiretroviral Drugs For Treating and Preventing HIV Infection in Kenya 2016**. In*.* Nairobi: Ministry of Health; 2016.

3. National AIDS and STI Control Programme: **Guidelines on the Use of Antiretroviral Drugs for Treating and Preventing HIV Infection in Kenya 2018 Edition**. In*.* Nairobi, Kenya: Ministry of Health; 2018: 210-210.

4. National AIDS Control Council: **Kenya HIV Estimates: Report 2018**. In*.* Nairobi, Kenya: Ministry of Health; 2018.

5. Irimu G, Ogero M, Mbevi G, Kariuki C, Gathara D, Akech S, Barasa E, Tsofa B, English M: **Tackling health professionals' strikes: An essential part of health system strengthening in Kenya**. *BMJ Global Health* 2018, **3**(6):1-5.

6. Njuguna J: **Impact of nurses’ strike in Kenya on number of fully immunized infants in 18 county referral hospitals**. *Journal of Health Care for the Poor and Underserved* 2018, **29**(4):1281-1287.

7. **Kenya's nurses strike takes its toll on health-care system**. *The Lancet* 2017, **389**(10087):2350.

8. Williams PCM: **The reality of the mortality statistics of the nurses’ strike in Kenya**. *The Lancet* 2017, **390**(10094):551.

9. National AIDS and STI Control Programme: **National Guidelines for PMTCT Peer Education and Psychosocial Support in Kenya: The Kenya Mentor Mother Program**. In*.* Nairobi, Kenya: Ministry of Health; 2012.

10. Larson BA, Bii M, Tsikhutsu I, Halim N, Wolfman V, Coakley P, Sugut W, Sawe F: **The Enhanced Mentor Mother ProgrAm ( EMMA ) for the prevention of mother-to- child transmission of HIV in Kenya : study protocol for a cluster randomized controlled trial**. *Trials* 2018, **19**:594-594.
